# The TOXIN knowledge graph: supporting animal-free risk assessment of cosmetics

**DOI:** 10.1093/database/baae121

**Published:** 2025-01-28

**Authors:** Sara Sepehri, Anja Heymans, Dinja De Win, Jan Maushagen, Audrey Sanctorum, Christophe Debruyne, Robim M Rodrigues, Joery De Kock, Vera Rogiers, Olga De Troyer, Tamara Vanhaecke

**Affiliations:** Department of In Vitro Toxicology and Dermato-Cosmetology (IVTD), Vrije Universiteit Brussel, Laarbeeklaan 103, Brussels 1090, Belgium; Department of In Vitro Toxicology and Dermato-Cosmetology (IVTD), Vrije Universiteit Brussel, Laarbeeklaan 103, Brussels 1090, Belgium; Department of In Vitro Toxicology and Dermato-Cosmetology (IVTD), Vrije Universiteit Brussel, Laarbeeklaan 103, Brussels 1090, Belgium; Department of In Vitro Toxicology and Dermato-Cosmetology (IVTD), Vrije Universiteit Brussel, Laarbeeklaan 103, Brussels 1090, Belgium; WISE lab, Department of Computer Science, Vrije Universiteit Brussel, Pleinlaan 9, Brussels 1050, Belgium; Montefiore Institute, University of Liège, Allée de la découverte 10, Liège 4000, Belgium; Department of In Vitro Toxicology and Dermato-Cosmetology (IVTD), Vrije Universiteit Brussel, Laarbeeklaan 103, Brussels 1090, Belgium; Department of In Vitro Toxicology and Dermato-Cosmetology (IVTD), Vrije Universiteit Brussel, Laarbeeklaan 103, Brussels 1090, Belgium; Department of In Vitro Toxicology and Dermato-Cosmetology (IVTD), Vrije Universiteit Brussel, Laarbeeklaan 103, Brussels 1090, Belgium; WISE lab, Department of Computer Science, Vrije Universiteit Brussel, Pleinlaan 9, Brussels 1050, Belgium; Department of In Vitro Toxicology and Dermato-Cosmetology (IVTD), Vrije Universiteit Brussel, Laarbeeklaan 103, Brussels 1090, Belgium

## Abstract

The European Union’s ban on animal testing for cosmetic products and their ingredients, combined with the lack of validated animal-free methods, poses challenges in evaluating their potential repeated-dose organ toxicity. To address this, innovative strategies like Next-Generation Risk Assessment (NGRA) are being explored, integrating historical animal data with new mechanistic insights from non-animal New Approach Methodologies (NAMs). This paper introduces the TOXIN knowledge graph (TOXIN KG), a tool designed to retrieve toxicological information on cosmetic ingredients, with a focus on liver-related data. TOXIN KG uses graph-structured semantic technology and integrates toxicological data through ontologies, ensuring interoperable representation. The primary data source is safety information on cosmetic ingredients from scientific opinions issued by the Scientific Committee on Consumer Safety between 2009 and 2019. The ToxRTool automates the reliability assessment of toxicity studies, while the Simplified Molecular Input Line Entry System (SMILES) notation standardizes chemical identification, enabling *in silico* prediction of repeated-dose toxicity via the implementation of the Organization for Economic Co-operation and Development Quantitative Structure–Activity Relationship Toolbox (OECD QSAR Toolbox). The ToXic Process Ontology, enriched with relevant biological repositories, is employed to represent toxicological concepts systematically. Search filters allow the identification of cosmetic compounds potentially linked to liver toxicity. Data visualization is achieved through Ontodia, a JavaScript library. TOXIN KG, filled with information for 88 cosmetic ingredients, allowed us to identify 53 compounds affecting at least one liver toxicity parameter in a 90-day repeated-dose animal study. For one compound, we illustrate how TOXIN KG links this observation to hepatic cholestasis as an adverse outcome. In an *ab initio* NGRA context, follow-up *in vitro* studies using human-based NAMs would be necessary to understand the compound’s biological activity and the molecular mechanism leading to the adverse effect. In summary, TOXIN KG emerges as a valuable tool for advancing the reusability of cosmetics safety data, providing knowledge in support of NAM-based hazard and risk assessments.

**Database URL**: https://toxin-search.netlify.app/

## Introduction

**Figure 1.**
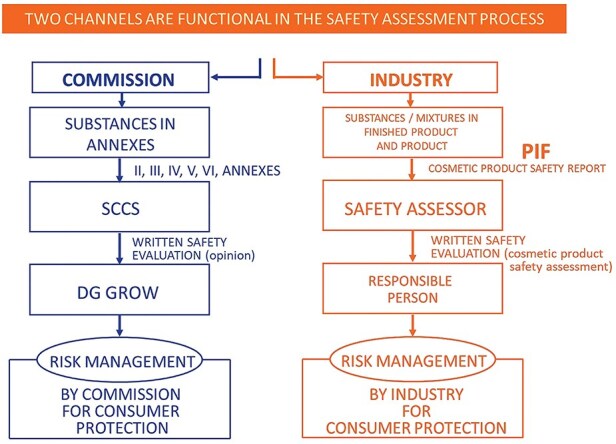
Taken from [1]. In the EU, cosmetic ingredient safety is ensured through two parallel and simultaneous control channels. Abbreviation: PIF, Product Information File.

Since July 2013, Regulation (EC) No. 1223/2009 has been fully enforced for cosmetic products within the European Union (EU). According to Article 3 of this regulation, any cosmetic product introduced to the EU market must be safe for human health under normal or reasonably anticipated conditions of use. To ensure the safety of cosmetic products, the safety of their composing ingredients is controlled via two parallel channels running simultaneously, as depicted in Fig. 1. At the EU level, the Scientific Committee on Consumer Safety (SCCS) evaluates cosmetic ingredients that could raise health concerns. These substances are listed in Annexes II–VI, with Annexes II and III serving as “negative lists”—List II includes prohibited pharmaceutical substances. At the same time, List III contains restricted substances allowed only for specific uses and concentrations, such as hair dyes. Annexes IV, V, and VI are “positive lists,” including approved colorants, preservatives, and ultraviolet (UV) filters. The SCCS provides comprehensive toxicological reports, known as “Scientific Opinions,” based on industry-prepared dossiers. These reports, which mainly include exposure data and toxicological studies, are used by the Directorate-General for risk management for the Internal Market, Industry, Entrepreneurship, and small and medium enterprises (SMEs) (DG GROW). All SCCS Opinions are publicly accessible through the EU’s official health webpage. Concurrently, the industry is responsible for the safety assessment of finished cosmetic products and their ingredients before market entry. Qualified safety assessors compile these assessments and report to the “Responsible Person,” the industry’s risk manager.

A significant complicating factor in the safety evaluation of cosmetics within the EU is that since March 2013, a total ban on animal testing has been in place [Regulation (EC) No. 1223/2009]. Before this date, a phased series of bans was implemented, starting with prohibiting testing cosmetic products on animals on 11 March 2004. This was succeeded on 11 March 2009, by the cessation of animal tests for all human health effects, except for long-term tests conducted outside the EU. Finally, a total ban for all toxicological endpoints, without any exceptions, was instituted on 11 March 2013. Consequently, only validated replacement alternative methods can be used to assess the safety of cosmetics. Validated replacement alternative methods are available for specific toxicity endpoints (local toxicity), such as skin and eye corrosion/irritation, skin sensitization, and phototoxicity. However, for more complex endpoints such as repeated-dose systemic toxicity, the development of New Approach Methodologies (NAMs) is necessary to replace traditional animal testing and make the transition toward animal-free risk assessment methodology without relying on experimental animals, commonly referred to as Next Generation Risk Assessment (NGRA) [1].

Because long-term and complex toxicological responses in living animals cannot be captured using single non-animal methods, it is necessary to combine several test methods for chemical hazard characterization, e.g. Integrated Approaches to Testing and Assessment (IATA). IATAs rely on an integrated analysis of existing information and the generation of new data using non-testing and testing methods. Non-testing methods involve *in silico* techniques like grouping (category formation based on toxicological criteria), read-across (extrapolating information from structural and functional analogs), and quantitative structure–activity relationship (QSAR) predictions. In the context of cosmetics, testing methods encompass *in chemico* approaches (providing physicochemical and mechanistic organic chemistry data) and *in vitro* and *ex vivo* methodologies that are preferably human-based and inform on the toxicological mechanisms of the compound under investigation[2–4].

This paper introduces a usable knowledge graph (KG), where ‘usability’ refers to the design of the KG to facilitate its use and integration into processes and applications, known as TOXIN knowledge graph (TOXIN KG), to assist in new risk assessment approaches for evaluating the safety of cosmetic ingredients. The developed TOXIN KG is built upon a graph-structured semantic technology, specifically the Resource Description Framework (RDF), and integrates relevant data using ontologies. The primary data source for TOXIN KG comprises existing safety data of annexed cosmetic ingredients, including information from animal studies carried out on cosmetic ingredients before the testing and marketing bans, as presented in SCCS scientific opinions. In addition, *in silico* toxicity predictions are made possible by integrating the OECD QSAR Toolbox into TOXIN KG. Furthermore, TOXIN KG can deduce implicit information from explicit data using rule-based mechanisms, exemplified for liver toxicity.

## Methodology

**Figure 2.**
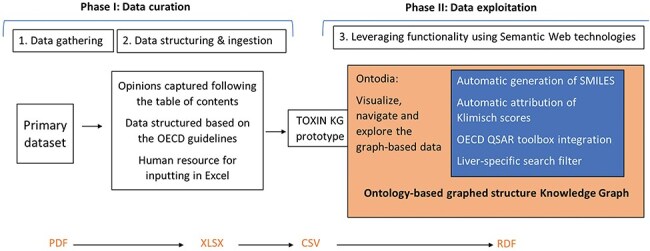
The development of TOXIN KG follows a structured workflow consisting of three main stages: data gathering, data structuring and ingestion, and leveraging functionality using Semantic Web technologies.

The development of the TOXIN KG prototype involves three main steps: (I) data gathering, (II) data structuring and input (In computer science, “data ingestion” refers to the process of data input, while in toxicology, the term “ingestion” specifically denotes oral intake. This paper has adopted a neutral term, “data input,” to prevent potential confusion.), and (III) leveraging the tool’s functionality (Fig. 2). The data processing workflow begins with the first step, data gathering, which involves collecting SCCS opinions in PDF format. During this phase, opinions not meeting initial criteria—specifically, the presence of a repeated dose toxicity (RDT) study and the absence of nano-ingredients—are excluded. The second step focuses on structuring the data by providing a framework based on OECD guidelines. This step ensures consistency through input rules and involves capturing and inputting data in a structured manner. As a resource-intensive phase, it relies on human expertise with a fundamental understanding of toxicology. Together, these two steps constitute the data curation phase.The third step prepares the curated data for exploitation by implementing tools for data retrieval, reliability assessment, and structural analysis. This step leverages the functionalities of the TOXIN KG and is closely tied to the data curation steps from the first two stages. It includes tasks such as automatic SMILES generation, automated assessment of toxicological studies using Klimisch scores, retrieving *in silico* HESS predictions for RDT, and utilizing profilers through the OECD QSAR Toolbox for identifying structural alerts and structure–activity relationship (SAR)–based chemical grouping. To enhance specificity, liver-specific search filters are introduced to refine the data. For improved data accessibility and user experience, Ontodia is employed to create an attractive and user-friendly interface.

### Phase I—data curation and transformation to a machine-processable format

Data curation, as defined by the National Library of Medicine, involves collecting, organizing, managing, and maintaining data to ensure its accuracy, reliability, and usability over time [5]. For TOXIN KG, this involves the following.

#### Data gathering

SCCS opinions from 2009 to 2019 without nano-ingredients and containing 90-day repeated-dose toxicity studies were used as sources [6]. These 93 opinions cover 88 cosmetic ingredients: 62 hair dyes, 9 preservatives and disinfectants, 5 UV filters, 2 fragrances, 2 solvents, 4 ingredients with multiple applications, and 4 substances with other cosmetic uses (oral hygiene, antiwrinkle, skin lightening, and hair-waving products). Each SCCS opinion details exposure, physicochemical properties, and toxicological studies. The opinions were downloaded in Portable Document Format (PDF) from the official EU health page for analysis [7].

The SCCS opinions often use natural language without a controlled vocabulary, posing a challenge for tools like TOXIN KG that rely on structured data. To address this, the Universe of Discourse for TOXIN KG has been meticulously structured at a granular level, facilitating the generation of the KG. Given the complexity and diversity of opinions, domain experts, rather than Artificial Intelligence (AI) technologies such as large language models (LLMs), were involved in interpreting these varied perspectives.

#### Data structuring and input

Data were manually entered into Microsoft Excel following predefined rules and the structure of the opinions. Excel was chosen for its simplicity and familiarity among toxicologists, allowing them to capture data without extensive Information Technology (IT) expertise. However, Excel has limitations in handling hierarchical data and large tables directly [8]. The Excel files were converted to a comma-separated value (CSV) format to facilitate data processing and transformation. CSV serves as an intermediary format that is more coding-friendly before converting the data into RDF. The CSV files were then transformed into RDF graphs using Relational Database (RDB) to RDF Mapping Language (R2RML). R2RML was employed because it is the standard mapping language for converting relational data into RDF. It is suitable for knowledge storage and semantic reasoning, especially in the context of linked data and the Semantic Web [9]. We adopted the R2RML-F engine for this process, which processes CSV files as in memory for the RDF generation process. This choice facilitates efficient transformation from the CSV format to RDF, leveraging the strengths of R2RML for mapping and the flexibility of the CSV format [10]. Storing data as RDF ensures interoperability by providing a standardized format that supports data integration and advanced querying. RDF organizes data for machine comprehension, allowing seamless data exchange between systems and improving the overall utility of the information. It also supports semantic applications, enhancing machines’ capability to reason about data and providing flexibility in data structuring [11].

##### Data structuring

**Figure 3.**
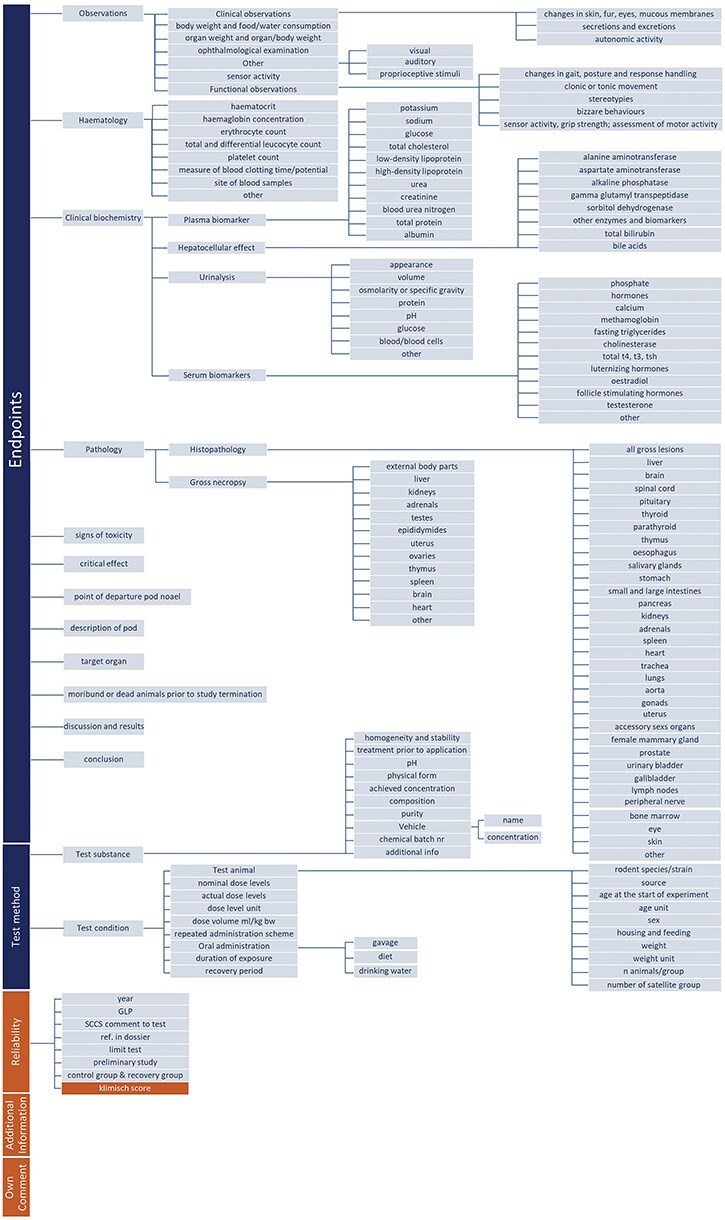
Data structure in TOXIN KG for OECD 408–guided RDT assessment, integrating liver-specific parameters, reliability metrics, and automated Klimisch scoring.

Data organization aligns with OECD guideline testing methods, breaking it into “concepts” and “value properties.” This process, termed “guideline profiling,” involves defining “concepts” as entities without data values that shape the data hierarchy and “value properties” as specific data values. In our data structure, concepts are denoted with capital letters, while value properties are written in lowercase. Initially, profiles were created for acute toxicity, RDT, toxicokinetics, and skin absorption. For example, the data structure for OECD test No. 408 on repeated-dose oral toxicity [12] (Fig. 3) includes standardized units such as ml/kg body weight (bw) for dose volume, and value types such as YES/NO for the value property “moribund or dead animals prior to study termination” [13]. This comprehensive structure supports automated Klimisch scoring and liver-specific search filters (see Phase II). The “Reliability” concept, created by the authors, was added to incorporate value properties capturing study credibility. This concept includes elements such as SCCS comments, study year, Good Laboratory Practice (GLP) status, ref. in dossier (links to the study reference mentioned in the opinion), and additional information fields. This structure facilitates future data queries using AI-based Natural Language Processing (NLP) tools.

Figure 3 illustrates the data structure utilized in TOXIN KG for assessing RDT in compliance with OECD test guideline No. 408. The structure includes key concepts such as clinical and functional observations, hematology, clinical biochemistry, and pathology in rodents exposed for 90 days. These are organized into three levels of concepts, each starting with a capital letter, and their value properties are aligned with OECD 408 requirements. Liver-specific parameters are integrated through the “hepatocellular effect” concept, which is delineated into specific value properties associated with liver enzymes and other biomarkers used to monitor liver function. For instance, the value property “moribund or dead animals prior to study termination” is mapped to Boolean values (YES/NO), while “mortality rate” provides textual explanations. The “Test animal” concept ensures clear representation by distinguishing numerical age values from their units (days, weeks, months and years). Additionally, the structure incorporates the “Reliability” concept, which includes value properties such as “additional information,” “own comments,” and “ref. in dossier.” These properties enhance data credibility and enable the automated assignment of Klimisch scores, a significant advantage of this data structure. The comprehensive design further supports liver-specific analysis by enabling the extension of liver-specific search filters and facilitating the exploitation of hepatotoxicity data (see Phase II).

##### Data input

**Figure 4.**
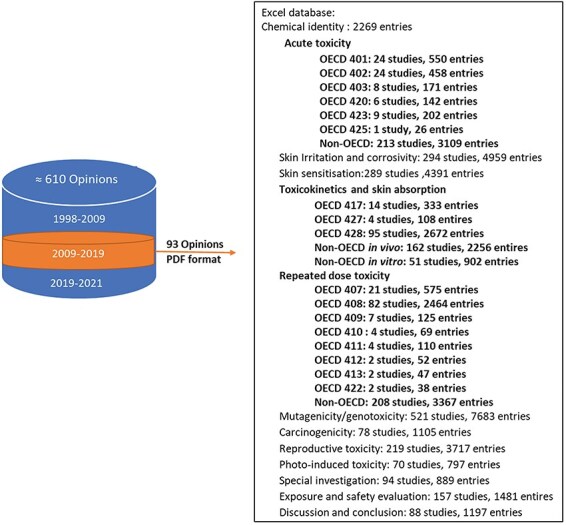
Overview of entries per toxicological end point and study, highlighting detailed data structures for key end points like repeated dose toxicity.

Initially, toxicology experts entered data into Excel, which was reviewed by another expert for accuracy. Although Excel was easy to use, it had limitations in capturing tables and structuring data hierarchy, leading to potential inconsistencies [8]. Tables were entered as linear text using specific predefined delimiters. However, Excel provided reliable data backup, making it a secure choice for initial data capture [14]. During data input, redundant information within a single study, such as repeated mentions of dosages, administration routes, and test substance specifications across different sections, was captured only once in TOXIN KG. This practice minimized unnecessary repetition and ensured a more concise and efficient data representation. Figure 4 provides an overview of the number of entries in the Excel files for each toxicological end point and study, regardless of their compliance with OECD guidelines, with human studies integrated to ensure that all relevant data are included in TOXIN KG. For key end points such as acute toxicity, RDT, toxicokinetics, and skin absorption, a detailed and comprehensive data structure was developed. This structure breaks down extensive text values into smaller, specific segments, enabling more effective data retrieval and enhancing the ability to query liver-specific effects and related information. In contrast, a less detailed data structure was implemented for other end points, resulting in larger, unsegmented text values. For ingredients with multiple opinions, data were extracted from various opinions, with conclusions drawn from the most recent one for accuracy. Later on, with LLM advancements, the TOXIN Report Analyzer (https://sccs-csv.netlify.app) using ChatGPT-4 was developed to extract text from SCCS opinions and convert it into tables automatically to speed up the data input process. Note that the generated tables are to be reviewed by experts to ensure accuracy (also see the Data governance section). This hybrid AI-expert approach ensures high-quality, scalable data processing and reproducibility [15].

### Data governance

Transitioning from human text-based data to computer-processable data underscores the crucial need for data accuracy and minimizing errors. The axiom “garbage in, garbage out” highlights the importance of precise data input for quality output. Fu *et al*. emphasized various dimensions of data governance: data accuracy, completeness, integrity, metadata management, availability, and authorization [16]. In TOXIN KG, toxicologists oversee data accuracy and completeness, while computer scientists handle metadata management, data availability, and authorization. Both groups share responsibility for data integrity. The quality assurance related to metadata management has been documented elsewhere [11]. Hence, only the other five remaining aspects are briefly discussed hereunder.

#### Data accuracy

Ensuring data accuracy and consistency is vital. Human errors in data reading, transferring, and inputting can compromise accuracy. We followed a rigorous data input process from each opinion, including a data validation step. Random sampling was conducted against the original SCCS opinions as the gold standard to refine this process further. For instance, in the case of repeated-dose toxicity studies, a total of 332 studies, we conducted random reviews on 10% of these studies (32 studies). The data input accuracy percentage was calculated by multiplying the number of correct entries (948) by 100 and dividing it by the total entries (990), resulting in an accuracy rate of 96%. Researchers may tolerate some errors, but tools for automated decision-making demand rigorous data governance to ensure accuracy. A computerized tool is yet to be implemented to verify and quantify data accuracy. To further enhance data accuracy and ensure the reliability of the information stored in the KG, toxicologists can input any comments or inconsistencies they may encounter during the data input phase, labeled as “own comments.” This feature contributes to data provenance by allowing experts to document observations and discrepancies, providing additional context and transparency for future data users.

#### Data completeness

Thoroughly capturing SCCS opinions prioritized data completeness. The “additional information” value property was introduced to capture supplementary details not explicitly requested by OECD guidelines. This ensures comprehensive data input and contributes to data provenance. An automated tool is yet to be implemented to verify and quantify data completeness.

#### Data integrity

Data integrity ensures accuracy, consistency, and completeness while preventing unauthorized modifications. It involves maintaining data quality and consistency over time [17]. Physical and logical integrity protects against disruptions, errors, and misuse. Physical integrity safeguards data storage, while logical integrity ensures consistency and security through entity, referential, domain, and user-defined rules. Key traits include completeness, accuracy, consistency, timeliness, and standards compliance. Mitigation includes limiting access, validating data, regular audits, error-detection software, and robust backups [18]. Human and transfer errors were elaborated on earlier under data accuracy. On the other hand, computer scientists need to tackle other issues endangering data integrity.

#### Data availability

SCCS opinions and integrated data, including TOXIN KG, are publicly accessible. A disclaimer notifies users that not all SCCS opinions are (yet) included in TOXIN KG.

#### Data authorization

User access to private and sensitive data is controlled through user privileges and predefined authorization policies. Toxicologists transcribe data but cannot generate RDF. Computer scientists have read-only access to Excel for RDF graph generation and maintain the ontology underlying TOXIN KG.

### Phase II—data exploitation

#### Leveraging the functionality of TOXIN KG by using Semantic Web technologies

Semantic Web technology enhances web content to be more meaningful and machine-understandable, improving information organization, linking, and representation. It adds explicit meaning to web content, enabling sophisticated processing and interpretation. Standardized data formats like ontologies and RDF define relationships between data elements. In predictive toxicology, semantic mapping and ontologies enable data integration, standardize representation, facilitate inference, and support knowledge sharing within the community, enhancing the accuracy and effectiveness of predictive models [19–22]. KGs, built using Semantic Web technology, organize information within a graph-like framework, linking entities (nodes) through relationships (edges). This arrangement enhances data analysis, retrieval, and decision-making capabilities. Integrating data involves amalgamating and streamlining diverse sources to construct a cohesive KG [23]. The primary data source for TOXIN KG is CSV files derived from the created Excel files and integrated into TOXIN KG using R2RML to ensure uniform and comprehensive integration. To make a thorough, multidata source tool for animal-free cosmetics’ hazard and risk assessment, we integrate data through semantic mapping, linking individual elements to a common ontology within TOXIN KG. ToXic Process Ontology (TXPO) was selected as the primary ontology structure [22], part of the Open Biomedical Ontologies Foundry. TXPO includes elements from Uber-anatomy ontology, Cell Ontology, National Center for Biotechnology Information (NCBI), Taxonomy, Chemical Entities of Biological Interest, Gene Ontology (GO), Phenotype and Trait Ontology, Integrating Network Objects with Hierarchies, Ontology of Genes and Genomes, and Disease Ontology [24–30]. TXPO focuses on toxicity mechanisms, particularly hepatotoxic mechanisms, aligning with TOXIN KG’s goals [31]. To enrich TXPO, we integrated ontologies such as GO, GO-Causal Activity Model (GO-CAM), and UniProt for human gene products [32].

Additionally, we incorporated biological pathway databases Kyoto Encyclopedia of Genes and Genomes (KEGG) and Reactome to provide a complete overview of biological processes, including biochemical reactions, substance transport, and gene expression [33–37]. The various datasets and ontologies have been integrated into the TOXIN KG by linking to external resources’ Internationalized Resource Identifiers (IRIs), resulting in a distributed KG. To maintain traceability and prevent incoherencies, these integrations are stored in separate “named graphs,” allowing users to easily manage and trace the origins of the information [38]. Sanctorum *et al*. detailed the automatic data integration approach by comparing labels and IRIs [39].

Four extra modules were developed to enhance TOXIN KG’s functionalities, focusing on improving the end-user experience through seamless integration of the enriched TXPO ontology.

##### Addition of SMILES to standardize chemical identification

Various molecular representations have been developed to accommodate the growing number of chemicals and meet computational needs. SCCS opinions use diverse string representations for cosmetic ingredients, including International Nomenclature of Cosmetic Ingredients (INCI) names, chemical names, trade names, synonyms, Chemical Abstracts Service (CAS) numbers, EC numbers, empirical formulas, and 2D graphics. These need standardization to avoid interoperability issues. SMILES, introduced by Weininger in 1988, provides a flexible and efficient chemical structure notation. We implemented SMILES annotations to ensure consistent representation across datasets, enabling accurate data retrieval and improving interoperability. While SMILES strings for small molecules can be easily written and are theoretically correct, variations can occur in generating SMILES models [40]. Canonical SMILES, produced through canonicalization algorithms, ensure a unique SMILES identifier [41, 42]. Including canonical SMILES in TOXIN KG is essential for providing a concise and standardized representation of chemical structures within linked data. Except for two polymer ingredients unsuitable for canonical SMILES notation, canonical SMILES has been manually integrated using sources such as PubChem, ChemSpider, and Cheminfo SMILES generator.

##### Automatic Klimisch score attribution to evaluate the reliability of studies

Toxicological studies must be reliable for safety evaluations, regulatory decisions, and public health protection. The Klimisch scoring system, introduced in 1997, assesses study reliability. A score of “1” means “reliable without restriction,” adhering to valid guidelines and often conducted under GLP. A score of “2” means “reliable with restriction,” while scores of “3” and “4” denote “not reliable” and “not assignable” due to flaws or lack of data [43, 44]. The ToxRTool, an Excel-based application developed by EURL ECVAM, enhances transparency when assigning Klimisch scores. It evaluates studies based on five criteria: (i) test substance identification, (ii) test organism characterization, (iii) study design description, (iv) study result documentation, and (v) design and result plausibility. Each criterion includes “normal” and “red” questions rated as 0 or 1. Achieving categories 1 or 2 requires a score of 1 for “red” questions [45]. Table 1 summarizes the ToxRTool criteria and their classification. We developed a JavaScript tool for Klimisch category assignment in TOXIN KG, focusing on OECD-compliant studies for acute and repeated-dose toxicity. Accurate Klimisch scoring relies on a comprehensive data structure to locate ToxRTool answers within TOXIN KG. For instance, to evaluate the question “Are sufficient details of the administration scheme given to judge the study?” (criteria Group iii, Question 6), essential details like dilution in diet, total volume applied, and media homogeneity must be retrieved. For the 90-day repeated oral toxicity test (OECD No. 408), the tool scans for “Test method: Test condition: Oral administration: gavage.” If found, it examines “Test method: Test substance: homogeneity and stability” or “Test method: Test condition: dose-volume ml/kg bw” (Fig. 3). This example highlights the detailed data structure designed following OECD guideline profiling, demonstrating the value added by this approach, as shown in Fig. 3. If the test substance is administered through diet or drinking water, the tool focuses on homogeneity and stability data, assigning a score of 0 or 1. For data related to positive and negative control groups (criteria Group iii, Question 4), the “Reliability” structure includes a “control group & recovery group” data block. This demonstrates the advantage of adding the “Reliability” concept by the authors—the tool checks for the presence of data here. If absent, it examines the dose level data for a “0” dose level representing the negative control. Studies following OECD guidelines generally expect Klimisch scores of 1 or 2, based on adherence to guidelines and GLP compliance. Some studies might omit properties like exposure duration and administration scheme. Therefore, we introduced default values to assume that missing details do not indicate non-compliance but are unintentional omissions. For OECD No. 408 (Fig. 3), default values include “repeated administration scheme” (assumed “7 days/week”) and “duration of exposure” (assumed “90 days”). These assumptions enable efficient evaluation of OECD-compliant studies and prevent erroneous 0 scores for red criteria questions.

**Table 1.** ToxRTool criteria and questions for Klimisch score grading

Criteria	Questions	Type of Questions (N: normal, R: red)
I: Test substance identification	Was the test substance identified? Is the purity of the substance given? Is information on the source/origin of the substance given? Is all information on the nature and/or physico-chemical properties of the test item given, which you deem indispensable for judging the data?	– R – N – N – N
II: Test organism characterization	Is the species given? Is the sex of the test organism given? Is information given on the strain of test animal plus, if considered necessary to judge the study, other specifications? Is age or body weight of the test organisms at the start of the study given? For repeated dose toxicity studies only: Is information given on the housing or feeding conditions?	– R – N – N – N – N
III: Study design description	Is the administration route given? Are doses administered or concentrations in application media given? Are frequency and duration of exposure as well as time-points of observations explained? Were negative and positive controls included? Is the number of animals per group given? Are sufficient details of the administration scheme given to judge the study? For inhalation studies and repeated dose toxicity studies only: Were achieved concentrations analytically verified or was stability of the test substance otherwise ensured is made plausible?	– R – R – R – R – R – N – N
IV: Study results documentation	Are the study endpoint(s) and their method(s) of determination clearly described? Is the description of the study results for all endpoints investigated transparent and complete? Are the statistical methods applied for data analysis given and applied in a transparent manner?	– N – N – N
V: Plausibility of study design and results	Is the study design chosen appropriate for obtaining the substance-specific data aimed at? Are the quantitative study results reliable?	– R – N

The five sets of criteria and their corresponding questions in the ToxRTool are presented. The third column specifies the weight assigned to each question, categorized as either “normal” (N) or “red” (R). To achieve reliability categories 1 or 2, questions marked as “Red” must be scored as 1.

##### Integration of OECD QSAR Toolbox profilers

In IATAs, *in silico* methods are indispensable for evaluating chemical toxicity. These methods connect chemical activity/properties with structural characteristics to predict activity data or physicochemical attributes. They also identify structural components influencing these properties within a mechanistic framework. In regulatory contexts, the credibility of *in silico* predictions is enhanced when integrated into a broader Weight-of-Evidence strategy, supported by other information like human-based *in vitro* data [46].

The QSAR Toolbox, developed by the OECD, is crucial for chemical hazard assessment [47]. It uses the (Quantitative) Structure–Activity Relationship/Structure–Property Relationship [(Q)SAR/SPR] methodology to predict chemical behavior by examining structural attributes and biological activities [48]. This toolbox includes software programs to profile, categorize, and address data gaps via (Q)SAR/SPR models and read-across techniques for different toxicological endpoints. The QSAR Toolbox encompasses 70 profilers in six groups: predefined, general mechanistic, endpoint–specific, empiric, toxicological, and custom. Metabolism profiling includes documented metabolism (400 observed pathways) and simulated metabolism, predicting the metabolic fate of target ingredients [49]. All these profilers are accessible within TOXIN KG by integrating the Application Programming Interface (API) of the QSAR Toolbox. The key profilers implemented in TOXIN KG are elaborated further.

###### Hazard Evaluation Support System

Hazard Evaluation Support System (HESS), in the toxicological profiler category, is relevant for repeated-dose liver toxicity. Developed by Japanese institutions and universities, it categorizes mechanistic insights into *in vivo* RDT for 500 chemicals, defining 33 categories across 14 toxicity types, including hepatotoxicity. Predictions are ranked based on the availability of toxicity mechanism information and a well-defined structural boundary [50]. Rankings are as follows:

“A”: Mechanism information present, clear structural boundary, reliable data gap filling.

“B”: Mechanism information present, unclear structural boundary, limited data gap filling.

“C”: No mechanism information, empirical tendency, potential for improvement.

###### Extended Cramer toxic hazard classification

In the general mechanistic category, the Cramer classification system, a structural classification system, typically assigns chemicals to three classes (I, II, and III) based on their anticipated levels of oral systemic toxicity. However, as recognized by the SCCS, this system is simplified into two classes by combining Classes II and III. This two-class system underpins the Threshold of Toxicological Concern (TTC) concept, primarily applicable to small quantities of chemicals, with exposure thresholds defined by the SCCS [1, 51, 52]. Chemicals with exposures below these TTC values pose a low risk of adverse health effects [53].

###### Documented and simulated metabolism profiler

This profiler provides pathways observed in mammals and simulations of *in vivo* and *in vitro* rat metabolism and skin metabolism. The skin metabolism simulator mimics mammalian liver metabolism [54].

It is important to note that the profilers provided in the QSAR Toolbox are not (Q)SAR models and have not undergone stringent validation to define applicability domains. Recent studies on profiler performance indicate limitations, showing that they may perform poor in predicting specific toxicological endpoints when tested against large datasets [55]. Therefore, in TOXIN KG, these profilers are used as an initial screening tool to identify potential toxicological alerts. For instance, for RDT, HESS predictions apply a similarity-based measurement focused on selected molecular features of the target chemical [56]. If the target chemical shows 50% structural similarity based on the Dice measure, it is assigned to the relevant toxicity alert. Thus, predictions from the OECD QSAR Toolbox API in TOXIN KG are only preliminary indicators rather than definitive toxicological assessments, which require a combination of more than one valid QSAR model and/or experimental validation.

##### Extended liver-specific filters for improved identification of hepatotoxicants

Although cosmetics are mainly for dermal application, the liver is the primary target organ after repeated oral exposure in laboratory animals [6, 57]. However, translating animal model findings to human outcomes is often flawed, as shown by drugs causing liver problems leading to market withdrawal [58–60]. Therefore, it is crucial to assess the human relevance of these observations using human-based NAMs. As a case study, we selected parameters based on literature and OECD guidelines for evaluating liver function disturbances. These include liver tissue damage through necropsy and histopathology, enzymatic changes [Alanine aminotransferase (ALT) (EC 2.6.1.2), aspartate aminotransferase (AST) (EC 2.6.1.1), alkaline phosphatase (ALP) (EC 3.1.3.1), gamma-glutamyl transpeptidase (GGT) (EC 2.3.2.1), sorbitol dehydrogenase (EC 1.1.1.14)], and biomarkers (total bilirubin, total cholesterol, fasting triglycerides, high-density lipoprotein, low-density lipoprotein, total protein, and albumin). These liver-specific search filters help identify cosmetics that may cause liver toxicity by highlighting observed harmful effects. The filters’ functionality is detailed in the Results and discussion section.

##### Enhancing data visualization and exploration via Ontodia

Efficient data presentation is essential for both human understanding and machine analysis. Humans and computers use different methods to grasp and establish connections within data. While text-based representation suffices for human comprehension, forming relationships between various entities can be challenging [30–33]. Graph databases offer an appealing solution to address the complexity of data understanding and interlinking, especially for computer processing. They provide superior query performance and flexibility, making them ideal for managing complex data. Ontodia (We have used a precompiled distribution of Ontodia that was made available on JSFiddle (https://jsfiddle.net/yn9ur13h/). This version, namely, 0.8.0, is hosted at https://unpkg.com/ontodia@0.8.0/dist/ontodia-full.min.js.) is a powerful tool for data representation [61]. It allows end-users to visualize and seamlessly explore graph data by interacting with RDF graphs [62]. In TOXIN KG, Ontodia’s utility is evident at two levels: at the information level through observations reported in the opinions and at the relationship level. The former links the same observations in TOXIN KG’s internal data, while the latter allows more profound indirect linkage between internal and linked data. Both levels are explained and illustrated in the “TOXIN KG links observations to toxicological mechanisms through ontologies” section. Ontodia significantly enhances data visualization and exploration within TOXIN KG, facilitating a comprehensive understanding and streamlined navigation of intricate information. Tools like Ontodia are “unaware” of the application domain; they only recognize that the KG encodes data using RDF and interacts with RDF via the SPARQL query language, another Semantic Web standard. The integration of Ontodia shows that end-users can engage with RDF in different ways and that using open standards facilitates the integration and development of tools on top of the KG.

## Results and discussion

The SCCS has issued scientific opinions since 1979, adapting their format and content to meet evolving legal requirements and scientific advances. On the EU health webpage, the opinions are organized by changes in the committee’s name: before October 2004 (“Scientific Committee on Cosmetology” and “Scientific Committee on Cosmetic Products and Non-Food Products”), October 2004 to March 2009 (“Scientific Committee on Consumer Products”), and April 2009 to March 2013, April 2013 to March 2016, April 2016 to December 2021, and beyond (“SCCS”). To enhance machine processability and simplify data organization, we classified the opinions into three periods, focusing on the 2009–19 period for creating the TOXIN KG (Fig. 4). Despite 220 opinions evaluating 163 chemicals within this timeframe, TOXIN KG development focused on 93 opinions on 88 cosmetic ingredients, excluding nanoingredients and including 90-day repeated-dose toxicity studies [6]. This limited dataset does not impede TOXIN KG’s development but presents an opportunity for expansion.

### Retrieving annexed cosmetic compound data with TOXIN KG

**Figure 5.**
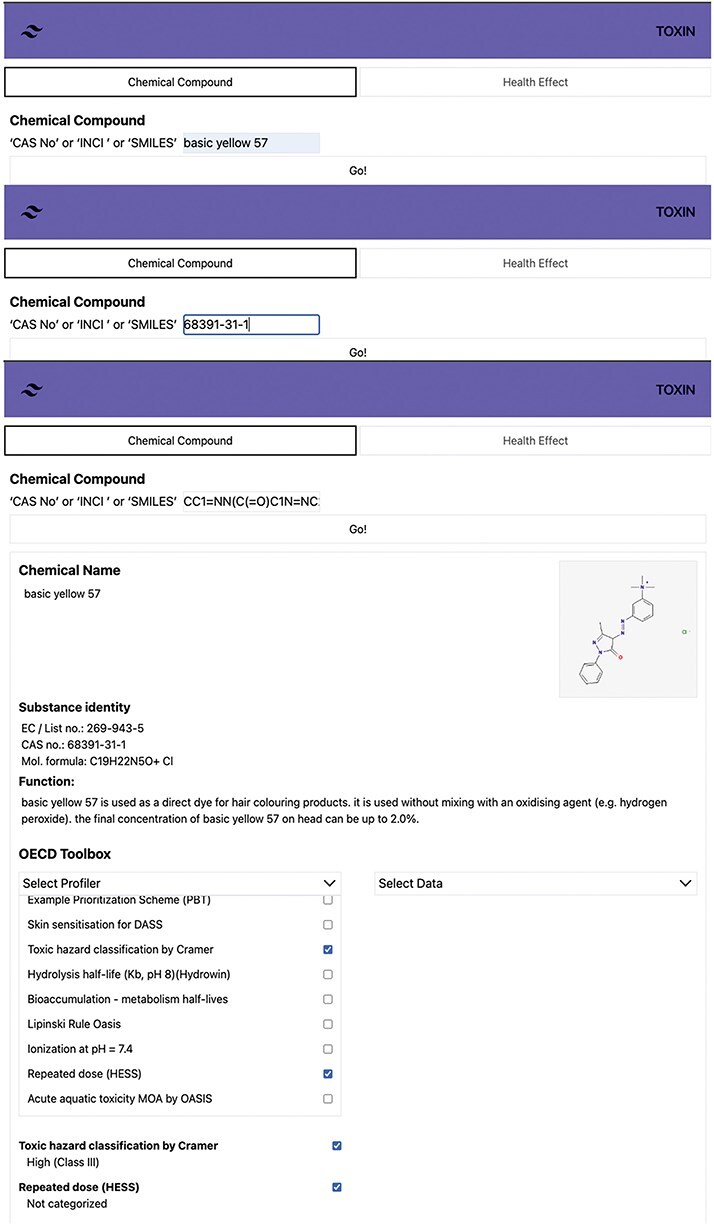
Search results for Basic Yellow 57 in TOXIN KG, providing detailed chemical information and access to 70 OECD QSAR Toolbox profilers for systemic and organ toxicity.

The user interface of TOXIN KG is designed according to user interface design principles to ensure human understandability [63, 64]. It offers functions to search and retrieve safety information about annexed cosmetic ingredients using CAS numbers, INCI names, or SMILES notations. The “Chemical compound” search option in TOXIN KG’s interface includes the OECD QSAR Toolbox profilers, providing insights into *in silico*-based hazard and metabolite predictions. Figure 5 shows the search results for “Basic Yellow 57” (CAS: 68391-31-1) and SMILES, providing detailed information about the chemical, including its identity, physicochemical properties, and intended use. Users can search for specific compounds using various identifiers, such as CAS number, INCI name, and SMILES. TOXIN KG integrates 70 OECD QSAR Toolbox profilers, with a focus on three particularly relevant to systemic and organ toxicity, which are accessible directly on the result page for convenient and efficient information retrieval [65, 66].

Switching the search function to “health effect” allows users to find compounds associated with specific toxicological effects. Results can be viewed in three formats: compound, opinion, or OECD. The compound view focuses on specific compounds, while the OECD view presents results per test guidelines. Maintaining uniform and standardized ingredient names is crucial for data retrieval. The EU health webpage shows slight variations in ingredient names, such as “HC Blue 15” appearing as “HC Blue No. 15” and “HC Blue No.15.” Such inconsistencies could lead to errors during data retrieval when using strict string matching. Alternative matching methods, like hard-set and proportional thresholds, offer solutions. Hard-set thresholds retain entities meeting specific criteria (e.g. “95% similarity”), while proportional thresholds retain a percentage of the best-performing entities regardless of absolute similarity. In TOXIN KG, proportional searching is in place; for instance, searching “yellow” will display every ingredient with “yellow” in its name [67]. In the first dataset, TOXIN KG addressed naming inconsistencies by manually incorporating canonical SMILES. Future developments will employ automatic SMILES generation and include data from remaining SCCS opinions. We propose a scalable method prioritizing precision over recall for evaluating correspondence between graphs [68–71].

### Automatic attribution of Klimisch scores to acute and repeated-dose toxicity studies

Data must be readily available in the system to create an automated scoring tool. Non-OECD studies present challenges due to documentation and study design issues. While SCCS comments are integrated into TOXIN KG’s reliability assessment part, the tool still needs to interpret these comments in text format. Therefore, the Klimisch scoring tool focuses solely on OECD-compliant studies, covering acute and RDT. To view the automatically scored studies, one can select either “acute toxicity” or “repeated-dose toxicity” under the “health effect” category and click “Go.” This will display a list of all studies related to the selected endpoint. Choosing the “OECD view” groups the studies according to their guideline.

**Figure 6.**
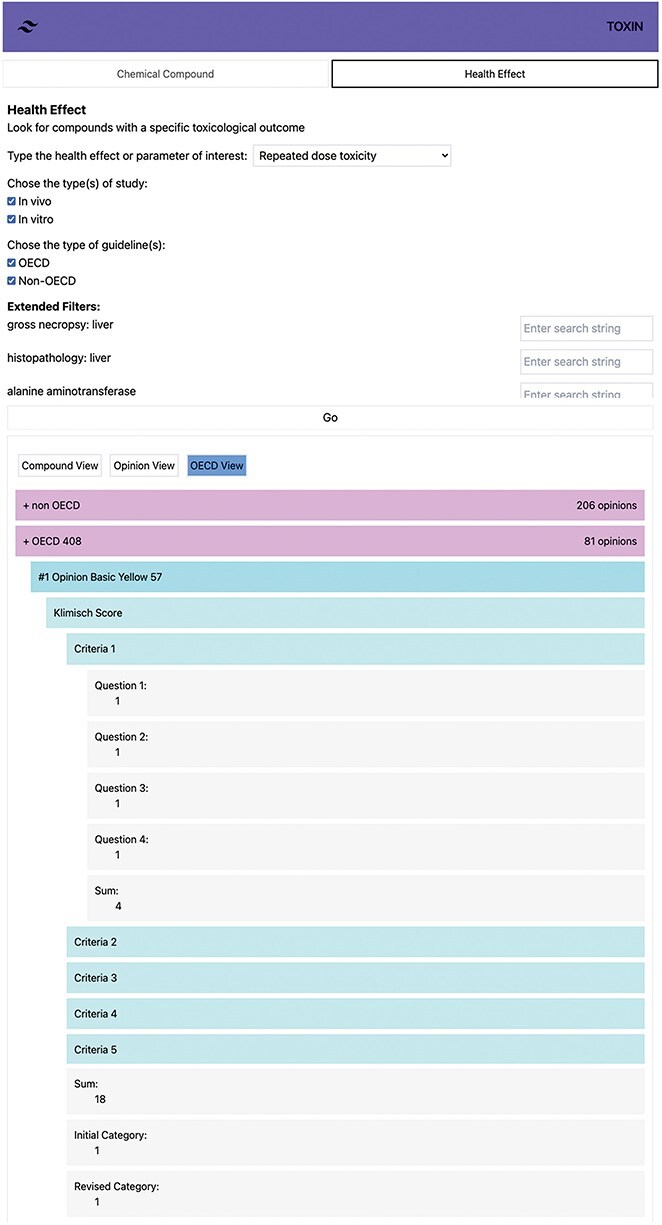
Automated Klimisch scoring of an OECD 408 study for Basic Yellow 57, showing total scores, categories, and individual criteria.

The ToxRTool calculates the initial category based on numerical points: a sum of 18 or higher falls into Category 1, 13 or higher belongs to Category 2, and <13 is Category 3. For the revised category, red criteria questions are first considered (Table 1). If all red criteria score 1, the study is Category 1; if not, it is Category 2 or 3, depending on the sum of points. Our tool uses the same reasoning as the ToxRTool. Figure 6 demonstrates the automated Klimisch scoring of an OECD 408 study for Basic Yellow 57, presenting the “sum,” “initial category,” “revised category,” and individual criteria scores. Users can access individual criteria scores by clicking on them for a detailed examination, with SCCS opinion data shown in black and default values in green within the TOXIN KG environment. In this instance, the tool assumes a repeated administration scheme of 7 days/week, which is necessary to assign a score of 1 to Criteria (iii), Question 3 concerning exposure frequency.

Including default values and compensating for missing critical attributes in the red criteria questions have proven to be practical in categorizing OECD-compliant studies as 1 or 2. Scoring studies using the automatic Klimisch system is complex due to limited detailed information in the opinions. An accuracy review was conducted by randomly selecting 20 out of 196 studies and comparing the automated scores with manual Klimisch scoring performed by domain experts. The initial accuracy was imperfect, leading to refined data pathways and better mimicking human scoring processes. This exercise highlighted the necessity of incorporating default values to ensure that critical attributes, particularly in red criteria questions, are appropriately addressed, thereby enhancing the accuracy of categorizing OECD-compliant studies into Category 1 or 2. Default values are based on the assumption that the study follows the guidelines. However, vital data, like the sex of animals, are sometimes buried within toxic effect descriptions. This is critical for determining Category 1 or 2 in the Klimisch scoring system. Despite these improvements, some challenges remain. For ingredients with comprehensive opinions, information like batch number or purity, initially stated in physicochemical properties, is not reiterated in the study report and is thus unavailable to the automatic scoring tool. To address this, default values are used to bridge information gaps. However, applying NLP techniques and LLMs could provide a more robust solution. NLP and LLM could automatically extract and interpret critical data points from the text, allowing for a more precise Klimisch scoring process. The challenge is greater for non-OECD studies. The absence of specific information affects the evaluation of all criteria, relying more on expert judgment. Although SCCS comments on these studies are available within TOXIN KG, NLP is essential to interpret these comments and assign Klimisch scores, ensuring a thorough and standardized evaluation of non-OECD studies.

### TOXIN KG identifies potential liver toxicants

To test the performance of the extended liver-specific search filters in TOXIN KG for querying potential liver toxicants, we manually screened the 93 opinions accounting for 88 different cosmetic ingredients for compound-induced changes in liver-related parameters in 90-day RDT studies. This analysis indicated that 53 ingredients altered at least one parameter possibly associated with liver toxicity in animals. Table 2 lists these 53 ingredients, their respective altered parameters, and their functions. However, replicating these results by a computer using extended filters is more complex. Navigating a knowledge base in toxicology presents challenges due to variations in terminologies used by different experts but with similar meanings. Traditional query systems that rely on keyword-based searches or structured query languages need help capturing these intricacies. We can identify three distinct forms of language complexity within the opinions. The SCCS employs terms like “similar to” and “comparable to” when assessing the likeness of a non-OECD study to an OECD guideline, creating ambiguity.

**Table 2.** The manually compiled Excel table, listing 53 potential liver toxicants from the first dataset of TOXIN KG

		Effects										
		*In vivo*										
Cosmetic compound	Function	Cholesterol	Triglycerides	ALT	AST	Phospholipids	Hepatic fat	Hepatocellular hypertrophy	Bilirubin	GGT	ALP	Hepatic necrosis
5-Amino-6-chloro-*o*-cresol	Precursor for hair dyeing products	Increase		Increase					Increase			
Acetylated vetiver oil—AVO = vetiveryl acetate	Fragrance	Increase		Increase								
Basic Brown 17	Direct dye	Increase	Increase		Increase					Increase		
Basic Red 51	Hair dye	Increase	Increase			Increase /decrease		Increase	Decrease	Increase	Increase	
Basic violet 2	Hair coloring agent	Increase			Decrease						Decrease	
*Bis*(butylbenzoate) diaminotriazine aminopropyltrisiloxane	UV filter			Different^*^	Different^*^							
Butylphenyl methylpropional	Fragrance	Decrease		Increase (non-OECD)				Increase (non-OECD)		Increase (non- OECD)		
Cetylpyridinium chloride	Desinfectant	Increase/decrease		Increase/decrease	Increase/decrease				Decrease		Decrease	
Decamethylcyclopentasiloxane (cyclopentasiloxane, D5)	Antistatic, emollient, humectant, solvent, viscosity controlling, and hair conditioning							Increase		Increase (inhalation)		
ecoG+	Preservative, packaging	Increase									Increase	
HC blue No. 15	Dye	Increase	Increase			Increase		Increase				
HC yellow No. 13	Hair coloring agent	Increase										
Hydroxyethyl-2-nitro-*p*-toluidine	Direct dye	Decrease		Increase	Increase				Increase		Increase	
Hydroxyethyl-3,4-methylenedioxyaniline HCl	Oxidative hair dye	Increase				Increase		Increase	Increase			
Hydroxyethyl-*p*-phenylenediamine sulfate	Oxidative hair coloring agent			Increase	Increase							
Hydroxypropyl *p*-phenylenediamine and its dihydrochloride salt (A165)	Oxidative agent	Increase	Increase	Increase	Increase				Increase	Increase		
Methylimidazoliumpropyl *p*-phenylenediamine HCl (A166)	Oxidative agent				Increase						Decrease	
*N*,*N*'-*Bis*-(2-hydroxyethyl)-2-nitro-*p*-phenylenediamine	Hair dye		Increase		Decrease							
*N*-Methyl-2-pyrrolidone	Solvent and surfactant	Increase/decrease^*^	Decrease								Decrease	
*o*-Aminophenol	Oxidative agent				Increase						Different	
Phenoxyethanol	Preservative	Decrease/increase	Increase^*^	Increase^*^^,^^§^	Decrease^*^^,^^§^	Decrease/increase				Increase^*^	Increase	
Toluene-2,5-diamine and its sulfate (COLIPA No. A5)	Oxidative hair coloring agent				Increase							
1-Hexyl 4,5-diamino pyrazole sulfate	Oxidative hair coloring agent								Increase			
2,6-Dihydroxyethylaminotoluene	Precursor for hair colors								Increase			
2,7-Naphthalenediol	Hair dye formulation								Increase	Increase		Increase
Citric acid (and) silver citrate	Preservative			Increase							Increase	
Diethylene glycol monoethyl ether	Solvent										Increase	
Methoxypropylamino cyclohexenylidene ethoxyethylcyanoacetate (S87)	UV filter								Increase			
Triclosan	Antibacterial	Increase (also^|^)/decrease	Increase/decrease	Increase	Increase/decrease^*^				Decrease^|^	Increase/decrease	Increase/decrease	Increase
Disperse black 9	Coloring agent			Increase	Increase					Increase	Increase	
HC red No.13	Direct dye			Decrease	Increase						Decrease	
Disperse blue 377	Hair dye		Decrease^*^^,^^§^	Increase	Increase				Increase			
Polysilicone-15	UV filter				Decrease ^i^				Decrease ^i^		Decrease ^i^	
*o*-Phenylphenol, sodium *o*-phenylphenate, and potassium *o*-phenylphenate	Preservative			Decrease^‖^							Increase	
Basic yellow 57	Direct dye			Increase					Increase			
HC red No. 7	Hair dye	Increase		Increase					Increase			
2-Hydroxyethylamino-5-nitroanisole	Semi-permanent hair dye			Change^¶^								
Acid orange 7	Direct dye								Increase^*^			
Basic red 76	Direct dye								Increase			
1,2,4-Trihydroxybenzene	Coloring agent								Increase^¶^			
Basic yellow 87	Dye								Decrease			
2,6-Diamino-3-((pyridin-3-yl)azo)pyridine	Direct dye	Altered							Altered			
2-Amino-4-hydroxyethylaminoanisole sulfate	Oxidative hair dye		Increase						Increase			
2-Amino-5-ethylphenol HCl	Oxidative hair coloring agent precursor								Increase			
HC yellow No. 9	Hair coloring	Increase	Increase									
Vitamin a	Antiwrinkle	Increase^|^										
Zinc pyrithione	Preservative	Increase^§^										
HC orange No. 6	Hair colorign agent	Increase										
Basic orange 31	Hair dye	Increase^‡^	Increase^‡^							Increase^‡^		
Acid red 92	Direct dye		Decrease	Decrease								
Disperse violet 1	Coloring agent	Increase^†^	Increase^†^									
Acid black 1	Coloring agent		Decrease									
HC blue No. 14	Coloring agent		Decrease									

The table also provides insights into the functions and *in vivo* effects related to liver parameters. It is important to note that the outcomes of these studies may exhibit contradictions due to various factors, such as differences in study design, sample size, methodology, or chance variations.

* Doubtful or not related to the test compound.

† Not supported histopathologically.

‡ Incidental.

§ Dermal.

| Human.

¶ Interference with the methodology.

‖ Only mentioned in SCCS conclusion and not in any cited study.

i Adaptive compensatory.

**Figure 7.**
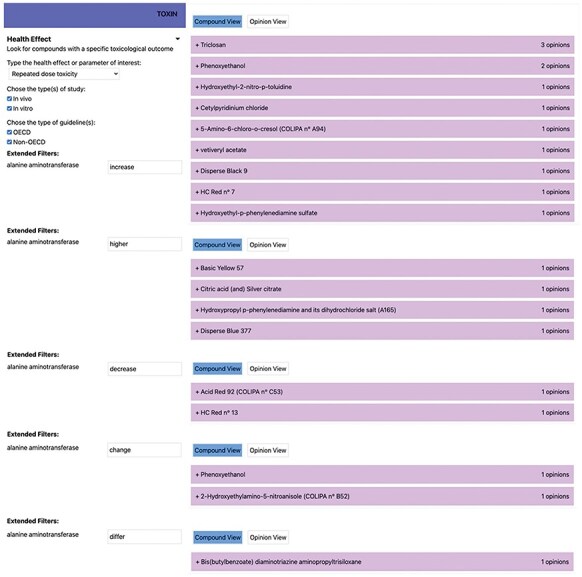
Displaying search results in TOXIN KG for ingredients that impact the ALT liver enzyme levels.

Additionally, toxic effects not caused by the test substance are described using terms like “incidental” and “doubtful.” Expressions like “no adverse” and “adaptive compensatory” also require clarification to ensure that the intended meaning is clear. From a scientific language perspective, terms such as “increase,” “raise,” and “higher” should be regarded as synonymous and interpreted accordingly when querying TOXIN KG. Variations in enzyme names also pose challenges; for example, “alanine aminotransferase” has been referred to as “serum glutamate-pyruvate transaminase,” with abbreviations like SGPT, GPT, ALAT, and ALT used in different opinions. TOXIN KG addresses these variations to ensure comprehensive search results comparable to those obtained manually using the Excel file containing the “raw data” of the SCCS opinions. Out of the 53 cosmetics identified as affecting at least one parameter related to liver toxicity in a 90-day RDT animal study, we manually found that 18 impact ALT levels. Figure 7 displays the search results in TOXIN KG for ingredients that impact ALT liver enzyme levels, showing how keywords such as “increase,” “higher,” and “decrease” are searched separately. Additionally, terms like “change,” “differ,” and “alter” are included to ensure a comprehensive search for ingredients reporting disturbances in ALT levels in animals. By addressing enzyme naming complexities and clarifying synonymous terms, TOXIN KG successfully retrieved all 18 relevant cosmetic ingredients affecting ALT levels, demonstrating its capability to match the accuracy of manual analysis.

Substantial enhancements can still be introduced to the search function by applying NLP and LLMs. Leveraging NLP can improve the tool’s understanding of user queries across diverse domains. NLP techniques such as Named Entity Recognition, Entity Linking, and Semantic Role Labeling can dissect user queries and extract pertinent information from opinions, thereby elevating the search results. Named Entity Recognition helps identify chemical compound names and essential terms, Entity Linking connects them to relevant database records, and Semantic Role Labeling assigns roles to words and breaks down user queries to understand the relationship between different parts. LLMs have also demonstrated their use in extracting information from text, interpreting texts, and translating questions in natural language to SPARQL queries [72]. While there have been significant advancements in NLP and AI-driven automated text analysis within the field of toxicology, additional fine-tuning is required before these technologies can be widely integrated into NGRA tools like TOXIN KG [73–76].

### TOXIN KG links observations to toxicological mechanisms through ontologies

In TOXIN KG, the enriched TXPO serves as the foundational framework, integrating data from various sources into a machine-friendly format and facilitating the exploration of toxicological data, including molecular pathways, cellular responses, and adverse outcomes [19]. TXPO’s explicit relationships simplify data navigation and enhance interoperability, fostering connections across toxicology. Recognizing the importance of identifying KG requirements through competency questions, TOXIN KG adheres to this practice [77]. The required information may originate from diverse sources, ideally through a semiautomatic extraction process. Linking entity descriptions to external representations enhances concept comprehension, even though errors in linking are considered noncritical [78]. TOXIN KG integrates information from external sources by establishing links to these sources while keeping integrations separate from internal data stored in dedicated entities known as “named graphs.” This strategic approach ensures traceability, enabling the identification of information origins [38].

**Figure 8.**
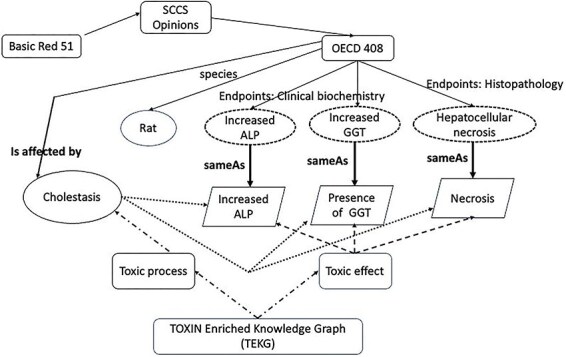
Relationships and features in TOXIN KG and TEKG for Basic Red 51, linking observations from OECD 408 tests to toxicological entities through direct and indirect relationships.

Figure 8 showcases insights obtained using semantic technologies and an enriched ontology. The information from a 90-day oral repeated-dose OECD 408 test for Basic Red 51, retrieved by TOXIN KG, is potentially linked to a liver-specific adverse outcome. The top section of the figure presents features retrieved from the SCCS opinion, including information about the test species and three key observations presented in dashed circles: increased ALP and GGT (from the clinical biochemistry end point) and hepatocellular necrosis (from histopathology). The bottom section of the figure includes entities from the TOXIN Enriched Knowledge Graph (TEKG), which integrates toxicological data from SCCS opinions with additional sources (enriched TXPO). These observations, “increased ALP,” “presence of GGT,” and “necrosis” are depicted in trapezoids and are linked to toxicological data through various relationships. Relationships are represented by arrows. Direct links are established in a “sameAs” relation between TOXIN KG and TEKG (solid lines), while dashed arrows indicate indirect links. Dashed lines symbolize indirect inheritance, and dotted lines denote indirect relations. Indirect inheritance allows flexible knowledge representation through intermediate classes, while indirect relations signify inferred connections not explicitly stated but deduced from the ontology structure [37]. The “sameAs” link and the “is affected by” link demonstrate linguistic-based rationale. “sameAs” indicates linguistic similarity, while “is affected by” links are inferred through reasoning, counting common effects between a disease and an opinion. These automated links offer insights into the likelihood that Basic Red 51 suggests liver cholestasis in the test subject. Ontologies and semantic technologies enable automation, while machine learning can also derive the “is affected by” relationship.

**Figure 9.**
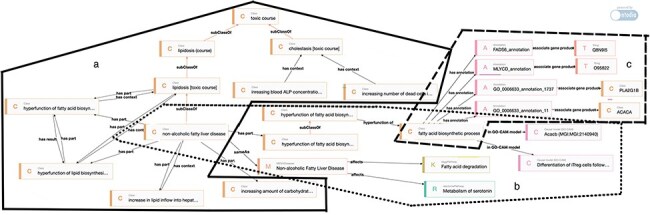
TEKG applications visualized through Ontodia, showcasing linked data to address competency questions on toxic processes, biological pathways, and compromised gene or protein functionality.

Figure 9, powered by Ontodia, introduces competency questions tailored to TOXIN KG, demonstrating how TEKG uses linked data to meet specific data retrieval needs and answer these questions. In Fig. 9, Zone a (solid line) illustrates the competency question: “Knowing some adverse effects observed in a subject, what diseases or toxic processes may affect this subject?” On the left, it depicts the relationships between toxic processes, adverse effects, and their interactions. Three key points are highlighted: “NAFLD” is categorized under “lipidosis,” indicating their connection. Two upward arrows represent adverse effects specific to NAFLD. Two hyperfunction symbols indicate adverse effects inherited by NAFLD from lipidosis, as NAFLD is a subtype of lipidosis. This illustrates the presence of these effects in NAFLD due to its classification. Additionally, the adverse effect “increasing blood ALP concentration” is linked to cholestasis using the “has context” predicate, while the “has part” predicate connects adverse effects to toxic processes, such as linking NAFLD to the hyperfunction of lipid biosynthesis. This approach enhances the organization and retrieval of information on specific adverse effects occurring within toxic events.

TEKG helps determine biological processes or pathways influenced by a disease. In Fig. 9, Zone b (dotted line) addresses the competency question: “What biological processes or pathways are impacted by a specific disease?” Here, NAFLD is linked to various pathways, including the “fatty acid biosynthetic process” and GO-CAM models, showing TEKG’s ability to identify affected biological processes. Zone c (dashed line) focuses on the question: “Which gene or protein’s functionality is hindered by a toxic process?” It depicts accessing gene products related to biological processes, such as acetyl-CoA carboxylase 1, involved in lipid metabolism. This gene represents a potential target for follow-up *in vitro* studies. [79–81].

Direct and indirect links are displayed interactively within the TOXIN KG environment, categorizing compounds affecting the same pathways based on *in vivo* data. For instance, “increased ALP” is common among eight ingredients, such as Basic Red 51 and phenoxyethanol (Table 2). Ontodia allows the grouping of these ingredients for further investigation, advancing SARs for liver toxicity [82–85]. The examples focusing on liver toxicity and related parameters can extend to other organs by broadening the search filters.

## Related work

In toxicology, TOXIN KG has been instrumental in developing an NGRA case study to thoroughly evaluate the liver toxicity of the hair dye HC Yellow No. 13 (HCY13). Historical *in vivo* data within TOXIN KG flagged HCY13 for its hepatotoxic potential (Table 2), while the *in silico* tools, including the OECD QSAR Toolbox and HESS toxicological profiler, identified a structural alert associated with fat accumulation (steatosis). Subsequent targeted *in vitro* tests with human hepatic-like cells assessed lipid metabolism-related markers and triglyceride accumulation, and a physiologically based pharmacokinetic model estimated internal liver concentrations of HCY13. These combined results confirmed that 2.5% HCY13 would not trigger liver steatosis under the assumed use conditions. Such case studies advance animal-free toxicity assessments and contribute to developing NGRA methodology. A detailed manuscript elaborating on these findings and methods is currently being prepared.

For KG generation, we adopted R2RML to transform CSV files into RDF using SQL, leveraging built-in SQL functions for data manipulation. At the time of writing, the RDF Mapping Language (RML) community, which proposes a “superset” of R2RML to transform any data into RDF, has started consolidating their efforts [86]. Although RML is still evolving and not yet a standard, tools like RMLWrapper (https://rml.io/tools/) and Morph-KGC (https://github.com/morph-kgc/morph-kgc) add complexity by requiring additional languages, such as Python. Given that R2RML meets our current needs, we chose to rely on this standard. Once RML is more stable, we may transition to an RML processor like BURP [87].

## Future directions

By integrating advanced AI-based tools and methodologies within TOXIN KG, we aim further to enhance data accuracy, completeness, and reliability, thereby supporting more robust and comprehensive toxicological assessments. At the start of the project in 2020, advanced AI tools like ChatGPT were yet to be available to replace manual text processes for data gathering, structuring, and input. As the field has rapidly evolved, several AI-based tools and methods are proposed for future implementation to enhance TOXIN KG’s capabilities. These include developing a pipeline using NLP and LLMs to automate data gathering from the EU health page, improving data capture efficiency and consistency, and addressing the manual effort needed due to the lack of harmonized naming systems [88].

The TOXIN Report Analyzer (under development) employs ChatGPT-4 to extract text from SCCS opinions and convert them into easy-to-understand tables. This process relies on incorporating precise prompts (specific instructions given to the AI model), guided by domain experts and guideline profiling, to ensure data accuracy and completeness, aligning with OECD guidelines. While AI aids in streamlining the process, domain experts will continue to review and correct the AI-generated tables to ensure accuracy [15, 89].

Automated consistency checks will also be implemented to improve data accuracy and completeness. AI tools will perform these checks at both the data extraction and input stages. Machine learning models will recognize patterns, validate data entries, and improve through expert corrections. Additionally, ontology-based tools will ensure semantic consistency by verifying that the data adhere to the structured format defined by the TXPO [90, 91].

In addition, a continuous feedback loop will be implemented with regular usability studies involving domain experts to refine TOXIN KG. These studies will assess navigation, ease of use, data accuracy, and efficiency across all interfaces. Experts will perform specific tasks using the different interfaces, with metrics like completion times, error rates, and user satisfaction measured. Postinteraction surveys and questionnaires will collect quantitative and qualitative feedback. Regular user feedback sessions will include structured interviews to discuss experiences, while analytics and user behavior tracking will identify patterns and difficulties, allowing continuous improvements to interface-based user feedback [92, 93].

## Conclusion

In conclusion, TOXIN KG is a multifaceted tool and gateway designed for ease of use. It offers access to existing cosmic historical animal safety data. It captures and annexes the wealth of knowledge embedded in SCCS opinions within a machine-processable graph format, obviating the need for intermediaries like gateways. This approach enables efficient *in silico* predictions and establishes a pivotal resource for exploring cosmetics-induced liver toxicity. Specifically, TOXIN KG helps identify and map biological pathways while elucidating the genes affected by toxic processes, laying the foundation for a deeper understanding of the intricacies involved. This knowledge is instrumental in guiding subsequent targeted *in vitro* studies utilizing human-based NAMs, thus contributing to generating novel mechanistic data.

## Data Availability

The data underlying this article are available in the article and in its online supplementary material.
